# Application of the ‘online assessment + FOBT at home’ to improve participation and the efficacy of opportunistic screening for colorectal cancer: a retrospective cohort study

**DOI:** 10.1186/s12889-023-17426-5

**Published:** 2023-12-18

**Authors:** Xudong Peng, Gang Tang, Yonghong Wang, Fanling Zeng, Yuedong Chen, Weidan Zhang, Chunmei Mo, Yana Yang, Shuang Li, Lian Bai, Dachun Xiao, Guolian Zheng, Wenhua Ran, Cheng Chen, Yonghong Yang, Yuanze Gao, Shuangwei Zhu, Zheng Huang, Dongqing Zhao, Chaofeng Wu, Li Xu, Zhengqiang Wei

**Affiliations:** 1 Gastrointestinal Surgical Unit, The First Affiliated Hospital of Chongqing Medical University, No. 1, Youyi Road, Yuzhong District, 400000 Chongqing, China; 2 Health Management Center, The First Affiliated Hospital of Chongqing Medical University, No. 1, Youyi Road, Yuzhong District, 400000 Chongqing, China; 3 Department of Gastrointestinal Surgery, People’s Hospital of Tongliang District, Chongqing, China; 4 Health Management Center, People’s Hospital of Tongliang District, Chongqing, China; 5 Gastrointestinal surgery Unit, Yongchuan hospital of Chongqing Medical university, Chongqing, China; 6 Health Management Department, Yongchuan Hospital of Chongqing Medical University, Chongqing, China; 7 Department of General Surgery, Qianjiang Central Hospital, Chongqing, Chongqing, China; 8 Department of Health Management, Qianjiang Central Hospital, Chongqing, Chongqing, China; 9 Anorectal Department, The Ninth People’s Hospital of Chongqing, Chongqing, China; 10 Gastroenteroanal Surgery, Fuling People’s Hospital Of Chongqing, Chongqing, China

**Keywords:** Colorectal cancer (CRC), Opportunistic screening, Social, Media, Fecal occult blood test (FOBT), Colonoscopy

## Abstract

**Background:**

Colorectal cancer (CRC) screening faces two major challenges: insufficient screening coverage and poor adherence. A smartphone applet named “Early Screening Assistant (ESA)” was developed to create an online risk-assessment and fecal occult blood test (FOBT) at home. This retrospective study was designed to evaluate whether the new CRC screening strategy can improve the colonoscopy participation rate (PR) and lesion detection rate (DR).

**Methods:**

In total, 6194 individuals who accepted normal health examinations and CRC screening based on the ESA from June 2020 to May 2022 were assigned to the ESA group. Accordingly, 7923 inhabitants who only accepted normal health examinations were assigned to the control group. The colonoscopy PR and neoplastic lesion DR were then compared between the two groups.

**Results:**

Overall, a higher proportion of subjects in the ESA group (285 of 6194 [4.6%]) completed colonoscopy than in the control group (126 of 7923, [1.6%]), *p* < 0.01). The neoplastic lesion DR also significantly increased in the ESA group (76 of 6194 [1.22%]) compared with the control group (15 of 7923 [0.19%]) (*p* < 0.01). The adjusted diagnostic sensitivity and specificity of the “Online assessment + FOBT at home” were 41.5% and 62.6% for neoplastic lesions, respectively.

**Conclusions:**

This retrospective cohort study confirmed that the new CRC screening strategy based on the “Online assessment + FOBT at home” can improve colonoscopy participation and the neoplastic lesion detection rate and may represent a promising screening strategy for CRC.

**Trial registration:**

This study was registered in *China Clinical Trial Registry* (https://www.chictr.org.cn*)* on 29/09/2022. Registration number: ChiCTR2200064186.

**Supplementary Information:**

The online version contains supplementary material available at 10.1186/s12889-023-17426-5.

## Background

The incidence of colorectal cancer (CRC) in China has risen steadily in recent years with the extension of life expectancy and Westernized dietary habits and lifestyles [1–3]. However, the survival rate of patients with CRC in China is significantly lower than that in developed countries such as South Korea and Australia [4]. Given the difficulties of primary prevention based on lifestyle changes and the limited effects of tertiary prevention based on standardized treatment, screening may be the most effective means with which to improve the prognosis of patients with CRC.

In 2012, China initiated the Cancer Screening Program in Urban China (CanSPUC) which targeted five types of cancer that are most prevalent in urban areas, including lung, breast and upper digestive tract cancer (esophageal and gastric cancers) and CRC [5]. The CanSPUC adopted a sequential CRC screening method and risk scores were calculated according to a risk assessment questionnaire. Although the CanSPUC is a free population-based cancer screening program organized by the government, many high-risk participants refused colonoscopy due to their fear of colonoscopy and a lack of knowledge about CRC. The colonoscopy participation rate (PR) was determined to be only 14% in high-risk participants and 1.85% in the whole population [6]. Opportunistic screening for CRC can also improve the prognosis of patients and reduce CRC-related incidence and mortality [7]. Compared with population-based screening strategies, opportunistic screening is not associated with a significant financial burden. The typical opportunistic screening strategy in China involves a risk questionnaire assessment and fecal occult blood test (FOBT); if either of these tests is positive, a colonoscopy is recommended. This strategy does not only save medical resources, it can also improve the PR of colonoscopy in high-risk groups [8].

In recent years, mobile health (mHealth) has been innovated rapidly, and involves telemedicine services, medical appointment management, Covid-19 prevention, and other medical applications [9]. mHealth has also been applied for cancer prevention. Kaitlin voigts key et al. piloted a Facebook-based social media referred to as #CRCFree to raise awareness for modifiable CRC risk factors. Analysis confirmed that #CRCFree increased the population’s healthy eating indices and reduced the diet infant index [10]. Lyson et al. designed an anonymous online platform named ‘Health Connect’ to share and discuss brief messages relating to the prevention of cervical cancer *via* Twitter. These authors found that HPV awareness could be increased through brief participation in this social media platform and the receipt of tailored health messages [11]. Similar studies have been reported for liver cancer [12] and colorectal cancer [13]. Although the use of mHealth in cancer management is currently limited to examinations, test reminders and health education, new applications including intervention measures are worth investigating.

In China, there is a lack of knowledge relating to CRC among residents, and few actively undergo CRC screening. Therefore, preventative knowledge and free screening for CRC was provided in health management centers to increase screening coverage. However, many residents are likely to be discouraged because of the complexity of the two FOBTs and the fear of colonoscopy. Based on the concept of mHealth, we developed a Wechat applet named Early Screening Assistant (ESA), which can realize a new screening strategy: Online risk assessment + FOBT at home. This new screening strategy involves (1) risk assessment online: a high-risk factor questionnaire (HRFQ) on the APP asks if subjects have CRC-related risk factors and (2) FOBT at home: subjects can use FOBT reagents to complete two FOBTs at home and then upload a photograph of their FOBT results through. The researchers then judged whether the subjects are at high risk of CRC based on their HRFQ and FOBT results and then send the preliminary screening outcome back to the subject *via* the ESA. This new screening strategy significantly improves the convenience of CRC screening, reduces screening costs, and due to a positive preliminary screening result, the acceptance of colonoscopy by patients may also increase.

“Online assessment + FOBT at home” has been applied to opportunistic CRC screening in seven health management centers over the last two years. Here, we retrospectively evaluate the effect of this screening strategy in improving the colonoscopy participation rate (PR) and neoplastic lesion detection rate (DR) in subjects undergoing health examinations.

## Methods

### Study population and design

We reviewed data from subjects aged 40 years and above who had undergone health examinations in seven health management centers from June 2020 to May 2022. These data include completion of the FOBT, the completion of colonoscopy and the results of colonoscopy. Subjects were excluded if (1) they had a prior history of CRC; (2) they had undergone colonoscopy within 5 years; (3) they had significant comorbidity that would pose a significant risk to the performance of colonoscopy; (4) they had participated in prior clinical trials related to CRC screening; (5) they had abnormal colonoscopy results but were unable to provide colonoscopy results; or (6) they were pregnant. All inhabitants were all recommended to complete medical examinations including colonoscopy, FOBT at hospital, rectal touch, and ‘Online assessment + FOBT at home’ based on the ESA, and so on. However, they can choose to do or not do them on their own. Individuals who accepted routine health examinations and CRC screening based on the ESA were assigned to the ESA group. Accordingly, subjects who only accepted routine health examinations were assigned to the control group. The main study outcome was colonoscopy PR, the secondary study outcome was neoplastic lesion DR.

### The early screening assistant

The “Early Screening Assistant” (ESA) WeChat applet features three main functions:

1. Risk assessment online

A high-risk factor questionnaire (HRFQ) asks subjects if they have CRC-related risk factors. Three primary risk factors includes: a family history of CRC, a family history of cancer and colorectal polyps. Five secondary risk factors includes: constipation, chronic diarrhea, mucus or bloody stool, a history of chronic appendicitis or appendectomy and a history of chronic cholecystitis or cholecystectomy.

(2) Photograph uploading function

After receiving a free FOBT bag containing two FOBT reagents (ABON Biopharm [Hangzhou] Co, Ltd) and an instruction card, subjects completed two FOBTs with an interval of approximately one week and then uploaded their FOBT photograph through the ESA. The colloidal gold method used in this FOB reagent has a detection sensitivity of 100ng/ml.

(3) Feedback function

The researchers judged whether the subjects were at high risk of CRC based on HRFQ and FOBT results, outcomes were then sent to the subjects via the ESA. Those with one primary risk factor or two secondary risk factors or a positive FOBT result were defined as a CRC high-risk population and were recommended to undergo colonoscopy as soon as possible [8, 14, 15]. Others (the general population) were recommended to undergo colonoscopy every 5–10 years. Feedback messages included contact information for colonoscopy appointments.

### Follow-up

**Fig. 1:**
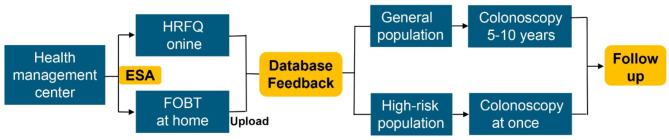
ESA screening flow chart

All subjects were followed up by telephone or WeChat. Subjects who reported normal colonoscopy results but were unable to provide colonoscopy results were recorded as being normal. If polyps had not been removed or pathological biopsy was not performed, the pathological type was judged by endoscopists. Advanced adenoma was defined as at least one adenoma ≥ 10 mm or at least one adenoma with villous components or at least three adenomas or high-grade neoplasia. (Fig. 1).

### Statistical analysis

Categorical data were analyzed by the χ2 test. Logistic regression analysis was used to identify the factors influencing the colonoscopy PR and neoplastic lesion DR. The selection bias of baseline data between the ESA group and control group was reduced by propensity score matching (PSM) at a ratio of 1:1. The propensity scores were estimated using a logistic regression model based on the following three variables: gender, age, and residence at hospital. The random number seed was set as 2,000,000 to ensure that the matching process could be repeated, and the match tolerance was set as 0.01. All data were analyzed using SPSS version 22.0 software.

## Results

### Colonoscopy PR and neoplastic lesion DR

**Fig. 2:**
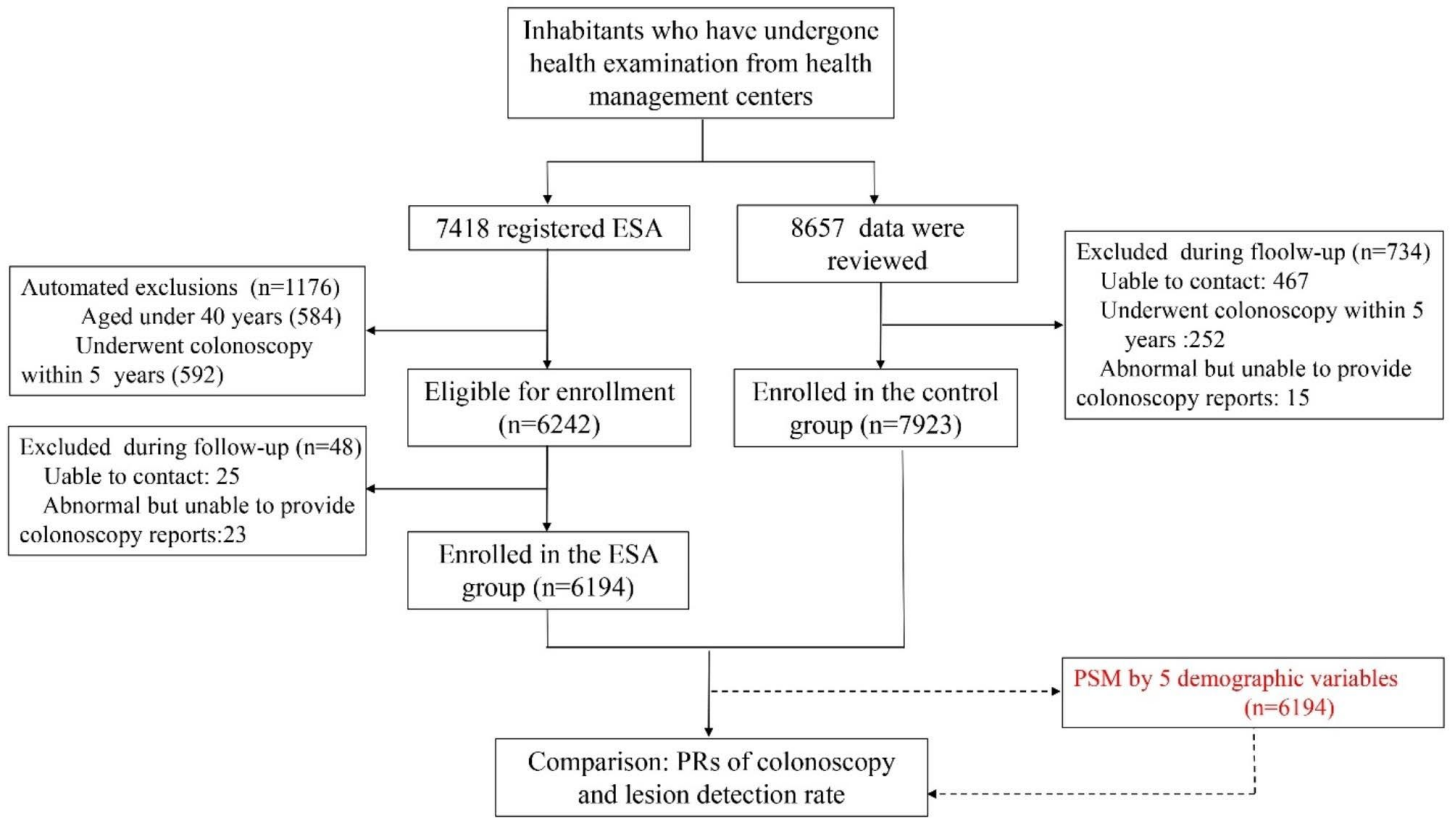
The subject selection process in this study

Finally, a total of 6194 inhabitants were enrolled in the ESA group and 7923 inhabitants were enrolled in the control group. A flow diagram is shown in Fig. 2. Compared with the control group, inhabitants in the ESA group were younger (*p* < 0.001) (Additional Table 1). In total, 53.4% of subjects completed at least one FOBT in the ESA group; this was significantly higher than the 0.6% of subjects in the control group. In total, 4.6% of subjects in the ESA group completed colonoscopy, this was significantly higher than the 1.66% of subjects in the control group. In the ESA group, there were 8 cases of CRC, 33 cases of advanced adenoma, 33 cases of common adenoma, and two cases of other tumors. In the control group, there was one case of colorectal cancer, three cases of high-risk adenoma and 11 cases of common adenoma. The neoplastic lesion detection rate in the ESA group was 1.22% (76/6194), which was significantly higher than the 0.19% in the control group (15/7923) (Table 1).

**Table 1:** Colonoscopy PR and neoplastic lesion DR

	ESA group n = 6194	Control group n = 7923	*P*-value
**FOBT participation**	3309	50	< 0.001
**Colonoscopy** **participation**	285	126	< 0.001
**Colorectal cancer**	8	1	0.013
**Advanced adenoma**	33	3	< 0.001
**Common adenoma**	33	11	< 0.001
**Other tumors**	2	0	0.192

PR, participation rate; DR, detection rate

### Factors associated with colonoscopy PR and neoplastic lesion DR

Univariate analysis was performed to identify factors associated with colonoscopy PR in the ESA group. Table 2 shows that the FOBT at home and positive HRFQ results were significantly associated with colonoscopy PR. Further multivariate regression analysis revealed that positive HRFQ and FOBT tests at home were both independent factors affecting colonoscopy PR (with odds ratios [ORs] of 2.6 and 8.0, respectively) (Table 2). Univariate analysis and multivariate logistic regression analysis showed that subjects who had a positive HRFQ result or completed the FOBT at home were more likely to have neoplastic lesions (ORs of 2.5 and 7.3, respectively) (Table 3).

**Table 2:** Univariate and multivariate analysis of factors associated with colonoscopy participation in the ESA group

	Colonoscopy participation			Multivariate analysis		
	YES	No	*P*-value	OR	95% CI	P-value
**Age**						
< 65	253	5350				
≥ 65	32	559	0.321	1.106	0.748–1.634	0.614
**Gender**						
Female	148	2971				
Male	137	2938	0.586	1.043	0.818–1.331	0.733
**Residence**						
Suburban	41	871				
Urban	244	5038	0.869	1.232	0.864–1.755	0.249
**Colonoscopy history**						
No	257	5565				
Yes	28	344	0.005	1.167	0.758–1.795	0.484
**Rectal touch**						
No	242	4988				
Yes	43	921	0.821	0.818	0.580–1.155	0.255
**FOBT** **at home**						
No	29	2870				
Yes	256	3039	< 0.001	8.013	5.431–11.823	< 0.001
**HRFQ**						
Negative	172	4815				
Positive	113	1094	< 0.001	2.606	2.013–3.374	< 0.001

FOBT, fecal occult blood test; HRFQ, high-risk factor questionnaire

**Table 3:** Univariate and multivariate analysis of factors associated with neoplastic lesion detection rate in the ESA group

	Neoplastic lesion			Multivariate analysis		
	YES	No	*P*-value	OR	95% CI	P-value
**Age**						
< 65	65	5538				
≥ 65	11	580	0.141	1.474	0.765–2.841	0.246
**Gender**						
Female	36	3163				
Male	40	3035	0.525	1.268	0.803–2.004	0.308
**Residence**						
Suburban	14	898				
Urban	62	5220	0.36	0.894	0.487–1.640	0.718
**Colonoscopy** **history**						
No	69	5753				
Yes	7	365	0.237	1.053	0.464–2.388	0.902
**Rectal touch**						
No	65	5165				
Yes	11	953	0.792	0.826	0.426-1.600	0.571
**FOBT** **at home**						
No	8	2891				
Yes	68	3227	< 0.001	7.294	3.492–15.239	< 0.001
**HRFQ**						
Negative	45	4942				
Positive	31	1176	< 0.001	2.524	1.564–4.073	< 0.001

FOBT, fecal occult blood test; HRFQ, high-risk factor questionnaire

### Sensitivity and specificity analysis

“Online assessment + FOBT at home” is a new CRC screening strategy; the efficacy of this strategy is unknown. 2,899 residents completed the online assessment, two FOBTs at home, of which 1,112 were defined as high-risk population and 1,787 were defined as common population. In the high-risk population, 224 received colonoscopy (20.1%), compared with 22 in the common population (1.2%). Data from 246 residents showed that the diagnostic sensitivity and specificity for colorectal disease were 92.3% and 11.1%, respectively. The diagnostic sensitivity and specificity for neoplastic lesions were 92.1% and 9.2%, respectively (Table 4). It is worth noting that the incidence of colorectal disease and neoplastic leisons in the commom population were as high as 54.5% and 22.7%, respectively.

**Table 4:** Diagnostic sensitivity and specificity of the “Online assessment + FOBT at home” strategy for colorectal desease and neoplastic lesions

Assessment	Colorectal disease		Neoplastic lesions	
	Yes	No	Yes	No
High-risk	144	80	58	166
Common	12	10	5	17
Total	156	90	63	183

Since the low PR of colonoscopy may lead to a increased sensitivity and decreased specificity, the adjusted sensitivity and specificity were calculated. Assuming that the incidence of colorectal disease and neoplastic lesions in residents who did not complete colonoscopy and residents who completed colonoscopy were equal, the sensitivity and specificity for colorectal disease were 42.3% and 67.2%, respectively. The diagnostic sensitivity and specificity for neoplastic lesions were 41.5% and 62.6%, respectively (Table 5).

**Table 5:** Adjusted diagnostic sensitivity and specificity of the “Online assessment + FOBT at home” strategy for colorectal desease and neoplastic lesions

Assessment	Colorectal disease			Neoplastic lesions	
	Yes	No		Yes	No
High-risk n = 1112	715	397		288	824
Common n = 1787	975	812		406	1381
Total	1690	1209		694	2205

### Colonoscopy PR and neoplastic lesion DR after PSM

After propensity score matching (PSM), 6194 participants were included in each group; the demographics of the two groups were similar (Additional Table 2). The colonoscopy PR (285/6194, 4.6%) and FOBT PR (3294/6194, 53.2%) in the ESA group were both higher than those in the control group (98/6194, 1.6% and 41/6194, 0.7%, respectively). The neoplastic lesion DR (75/6194, 1.21%) and colorectal desease DR (185/6194, 3%) in the ESA group was also significantly higher than those in the control group (7/6194, 0.01% and 52/6194, 0.8%) (Table 6).

**Table 6:** Colonoscopy participation rate and lesion neoplastic lesion detection rate after PSM

	ESA group		Control group		*P*-value
	Yes	No	Yes	No	
Colonoscopy participation	285	5909	98	6096	< 0.001
FOBT participation	3295	2899	41	6153	< 0.001
Neoplastic leision	75	6119	7	6187	< 0.001
Colorectal desease	185	6009	52	6142	< 0.001

## Discussion

Although CRC screening technologies such as FOBT, the CEA test, stool DNA testing, and colonoscopy have been widely used, CRC screening still faces two major challenges: insufficient screening numbers and poor adherence [16]. In CanSPUC, the colonoscopy PR in the overall population was only 1.85%, even though this program provides free colonoscopy to high-risk populations. It is thus important to identify methods that can mobilize more people to participate in and complete screening.

This study set up screening counters in health management centers and promoted offline and online information by the Wechat applet. We aimed to offer guidance to those with poor knowledge of CRC and to those with no obvious symptoms but who have health management needs. In contrast to traditional popular science awareness methods, such as newspapers and books, social media has a wide dissemination range and represents a rapid and targeted broadcast with low costs. In addition to obtaining FOBT reagents in health management centers, the subjects in different regions could also obtain free reagents by mail, thus extending the coverage of this strategy. Unlike reported strategies that adopted financial incentives, including direct monetary incentives and a lottery to increase CRC intake [17, 18], screening based on social media does not require significant financial support and appears to be sustainable.

To increase the accuracy, CRC preliminary screening requires subjects to complete two FOBTs, thus making screening more difficult. In general, there are no financial incentives or full-time employees designated to collect samples. If subjects must go to medical institutions for two FOBTs, many of them will be discouraged. Based on this background, this study proposed a screening strategy known as “FOBT at home”. Subjects conducted FOBTs at home according to a specific instruction card and video on the ESA; then, the subjects uploaded test photographs to researchers via this applet. Compared with the control group in this study, the PR of FOBT in the ESA group was significantly higher (by 84.7-fold). This data revealed an obvious advantage over previously reported strategies [19, 20]. Our data suggest that this new screening strategy may significantly improve adherence to CRC opportunistic screening.

Colonoscopy is the gold standard for diagnosing CRC and precancerous diseases. People over the age of 40 years need to undergo colonoscopy every 5–10 years. This study showed that subjects who completed the FOBT at home or had positive HRFQ results were more likely to accept colonoscopy (8 and 2.6-fold more likely, respectively). In other words, although our study did not directly subsidize the cost of colonoscopy, the increased participation in FOBT and HRFQ contributed to an overall improvement in the colonoscopy PR. Previous studies have reported that patient navigation could increase the screening intake via three approaches: (1) increasing awareness of the necessity for screening; (2) helping patients to overcome the fear of colonoscopy, and (3) providing convenient clinical services for colonoscopy appointments, disease treatment and follow-up [21, 22]. In this study, when feeding back the preliminary screening results (FOBT and HRFQ) to the subjects who participated in the ESA, we also provided the contact information of navigators for counseling and colonoscopy appointments. This practice may have improved the colonoscopy PR.

The carcinogenesis of colorectal polyps generally takes 5–10 years. The significance of screening is to detect precancerous lesions and early cancers. In this study, the overall DR of neoplastic lesions in the ESA group was 1.2%; this was 6.74-fold higher than that in the control group. The adenoma detection rate (ADR) of colonoscopy is regarded as a primary benchmark of colonoscopy. In all subjects in the ESA group who underwent colonoscopy, the ADR was 25.96%; this was significantly higher than the 11.49% in the CanSPUC program and also reached the standard recommended by the American College of Gastroenterology and the American Society for Gastrointestinal Endoscopy (25%) [23]. Sensitivity and specificity are other measures used to judge the efficacy of screening tools. In most CRC screening programs, subjects received free FOB reagents in the mail; then, the subjects collected and mailed samples of feces to the laboratory for testing to insure accuracy [24, 25].

In the ESA group, the diagnostic sensitivity of the risk assessment online and FOBT at home for neoplastic lesions was 92.1%, however, the diagnostic specificity of this screening strategy for neoplastic lesions was only 9.2%. While it may not be feasible for all subjects to undergo a colonoscopy, out of the total 6,194 subjects who completed the ESA assessment in this study, only 246 finished two FOBTs at home and received colonoscopy. Assuming that the incidence of colorectal disease and neoplastic lesions in the residents who did not undergo colonoscopy is equivalent to those who underwent colonoscopy, the sensitivity for colorectal disease would be 42.3%, and specificity 67.2%; for Neoplastic lesion, sensitivity is 41.5%, and specificity 62.6%. It is important to acknowledge that current sensitivity and specificity data may be subject to bias due to the limited sample size. The incidence of colorectal disease and neoplastic lesions in the common population was also high, with rates as high as 54.5% and 22.7%, respectively, indicating a potential for missed diagnoses in CRC screening utilizing “Online assessment + FOBT at home”. Anyway, high sensitivity and low specificity could be potential disadvantage of this strategy in that it could increase the cost of colonoscopy and the risk of potential colonoscopy-related complications. In future, the cost-effectiveness of this strategy for CRC screening needs to be evaluated further by health economic analysis.

Covid-19 broke out in late 2020 and affected almost all countries. In addition to causing direct damage to the medical system, this virus also exerted significant effects on basic health services, including cancer screening [26]. Systematic screening generally requires the gathering of subjects; however, maintaining social distance is an important aspect of controlling epidemic disease. By using the ESA, subjects can conduct risk assessment on mobile phones at any location and complete two FOBTs at home; they can then undergo colonoscopy at a nearby hospital if necessary. This screening strategy is low-cost and minimizes the flow of subjects; this may represent a suitable screening mode in the context of the Covid-19 pandemic.

### Limitations

The control group was selected from all residents who accepted routine health examinations, ensuring its representativeness across the seven health management centers. Individuals who accepted routine health examinations and CRC screening based on the ESA were assigned to the ESA group. Consequently, there is a possibility that some individuals interested in CRC screening were included in the ESA group. However, determining whether the increased CRC screening PR in the ESA group is attributable to applet functions or enrollment bias remains challenging. Future investigations should aim to mitigate inherent biases between the ESA and control groups through an RCT design.

## Conclusions

The “Online assessment + FOBT at home” strategy focuses on solving three problems faced by CRC screening in China: (1) the high cost of human resources and small coverage, (2) sampling difficulties related to the FOBT, and (3) low screening adherence. This study reviewed two years of data and confirmed that the screening mode can significantly improve the PR of colonoscopy and the lesion DR; however, the specificity is insufficient. Whether it can be widely used in CRC screening needs to be investigated from a health economics point-of-view. To the best of our knowledge, the ESA is the first mHealth to be used for CRC screening that combines health education and intervention.

### Electronic supplementary material

Below is the link to the electronic supplementary material.

Supplementary Material 1

Supplementary Material 2

## Data Availability

The datasets used and/or analyzed during the current study are available from the corresponding author on reasonable request.

## Abbreviations

CRC: Colorectal cancer
ESA: Early Screening Assistant
FOBT: Fecal occult blood test
PR: Participation rate
DR: Detection rate
CanSPUC: Cancer Screening Program in Urban China
mHealth: Mobile health
HRFQ: High-risk factor questionnaire
PSM: Propensity score matching
