# Supplementary material for: Application of the ‘online assessment + FOBT at home’ to improve participation and the efficacy of opportunistic screening for colorectal cancer: a retrospective cohort study
Source: BMC Public Health. 2023 Dec 18;23:2534. doi: 10.1186/s12889-023-17426-5 (PMC10729553; doi:10.1186/s12889-023-17426-5)
Supplement: Supplementary file 1 — Supplementary Material 1 [file 12889_2023_17426_MOESM1_ESM.docx]

Additional table1. The baseline demographic data of two groups

| **Demographic data** | **ESA group**  **n=6194** | **Control group**  **n=7923** | ***P*-value** |
| --- | --- | --- | --- |
| **Age** |  |  | <0.001 |
| ≥65 | 591 | 1019 |  |
| <65 | 5603 | 6904 |  |
| **Gender** |  |  | 0.418 |
| Male | 3075 | 3879 |  |
| Female | 3119 | 4044 |  |
| **Residence** |  |  | 0.486 |
| Urban city area | 5282 | 6723 |  |
| Suburban County | 912 | 1200 |  |

Compared with the control group, inhabitants in the ESA group were younger.
