# Supplementary material for: Application of the ‘online assessment + FOBT at home’ to improve participation and the efficacy of opportunistic screening for colorectal cancer: a retrospective cohort study
Source: BMC Public Health. 2023 Dec 18;23:2534. doi: 10.1186/s12889-023-17426-5 (PMC10729553; doi:10.1186/s12889-023-17426-5)
Supplement: Supplementary file 2 — Supplementary Material 2 [file 12889_2023_17426_MOESM2_ESM.docx]

Additional table2. The baseline demographic data of two groups after PSM

|  | ESA group | Control group |
| --- | --- | --- |
|  | 6194 | 6194 |
| Age |  |  |
| ≥65 | 591 | 591 |
| <65 | 5603 | 5603 |
| Gender |  |  |
| Male | 3075 | 3039 |
| Female | 3119 | 3155 |
| Residence |  |  |
| Urban city area | 5282 | 5269 |
| Suburban County | 912 | 898 |

The demographics of the ESA group and control were similar after PSM.
